# Right Ventricular Subclinical Dysfunction as a Predictor of Postoperative Adverse Clinical Outcomes in Patients with Femoral Fracture

**DOI:** 10.3390/jpm14070673

**Published:** 2024-06-22

**Authors:** Hyun-Jin Kim, Hyun-Sun Kim, Jeong-Heon Heo

**Affiliations:** 1 Division of Cardiology, Department of Internal Medicine, Hanyang University College of Medicine, Hanyang University Guri Hospital, 153 Gyeongchun-ro, Guri 11923, Republic of Korea; 2 Department of Nursing, College of Nursing, Eulji University, 712 Dongil-ro, Uijeongbu 11759, Republic of Korea

**Keywords:** echocardiography, femoral fracture, pneumonia, pulmonary oedema, pulmonary thromboembolism, right ventricle

## Abstract

Background: Femoral fractures often lead to complications such as altered pulmonary hemodynamics. Right ventricular global longitudinal strain (RV GLS), which correlates with pulmonary hemodynamics, indicates the subclinical function of the right ventricle (RV). This study aimed to investigate the predictive value of RV GLS for the risk of adverse clinical composite outcomes in patients with femoral fractures. Methods: Data were obtained from a prospective single-center cohort of patients hospitalized for femoral fractures and followed up for at least 1 year between March 2021 and October 2022. The primary outcome was the development of an adverse composite clinical event, which included pneumonia, pulmonary oedema or effusion, pulmonary thromboembolism, and all-cause mortality within the 1-year period following surgery. Results: Among the 163 patients, 36 (22.09%) experienced adverse composite clinical events during 1-year follow-up. The adverse outcome group demonstrated poorer RV GLS and RV free wall strain values than the non-adverse outcome group. The optimal cut-off value of RV GLS for predicting composite adverse clinical events was −12.55%. The cumulative composite event-free survival rate was significantly lower in the RV GLS ≥ −12.55% group (log-rank *p*-value = 0.003). After adjusting for confounding factors, multivariate Cox proportional hazards regression analyses showed that RV GLS ≥ −12.55% independently increased the risk of composite adverse clinical events by 2.65-fold. Conclusions: Poor RV GLS is a significant predictor of adverse clinical outcomes in patients with femoral fractures. Specifically, an RV GLS value of ≥ −12.55% indicated a substantially increased risk of adverse events.

## 1. Introduction

Femoral fractures, typically resulting from high-energy trauma or osteoporosis in older adults, are a significant public health concern owing to their frequency and associated complications [1,2]. These fractures are not only a leading cause of hospitalization but also carry a high risk of mortality, particularly in older populations. Complications, such as pneumonia, pulmonary oedema or effusion, and pulmonary thromboembolism, can worsen the prognosis of these patients [3,4,5]. In long bone fractures, such as femur fractures, the release of bone marrow and fat emboli into the bloodstream is a significant concern [6,7]. These emboli can travel to the pulmonary circulation, posing risks to pulmonary function and right ventricular (RV) performance [7,8]. The physiology of the RV, which handles lower pressures and is more compliant than the left ventricle, makes it particularly susceptible to increased strain from such embolic events [9]. Moreover, the impact of such fractures on cardiac function, particularly in the RV, is an area of emerging interest. RV strain evaluated using transthoracic echocardiography (TTE) is increasingly recognized as a vital indicator of subclinical RV dysfunction [10]. However, the predictive value of the RV longitudinal strain for femoral fractures remains unclear. We hypothesize that diminished RV longitudinal strain is a significant predictor of adverse clinical outcomes in patients with femoral fractures.

This study aimed to investigate the predictive value of RV longitudinal strain, as determined by TTE, for the risk of 1-year adverse clinical composite outcomes, including pneumonia, pulmonary oedema or effusion, pulmonary thromboembolism, and all-cause mortality, in patients diagnosed with femoral fractures.

## 2. Materials and Methods

### 2.1. Study Design and Population

**Figure 1:**
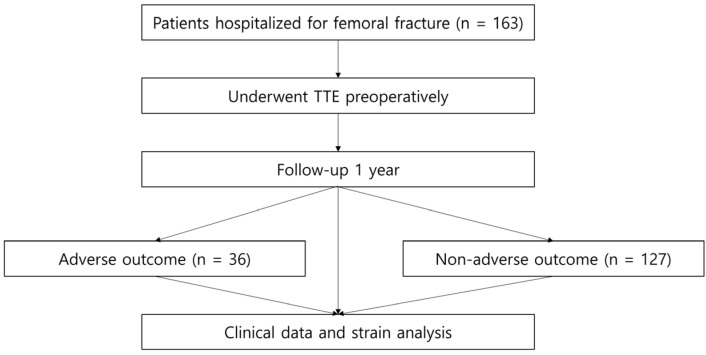
Study flow diagram. TTE, transthoracic echocardiography.

The study data were obtained from a prospective single-center cohort of patients hospitalized for acute femoral fracture and followed-up for at least 1 year after discharge. From March 2021 to October 2022, 163 patients diagnosed with femoral fracture at the time of admission or with signs or symptoms of femoral fracture that were confirmed through conclusively established imaging tests such as radiography and computed tomography (CT) were included (Figure 1). The diagnosis of femoral fracture was confirmed preoperatively, and all patients underwent TTE. A minimum of 1-year of follow-up was strongly recommended for all patients, and outcome data from regular visits were recorded prospectively. The 163 enrolled patients with femoral fracture were classified into two groups according to the development of adverse clinical outcomes during follow-up: an adverse outcome group and a non-adverse outcome group. The non-adverse outcome group included patients who did not experience adverse clinical outcomes during the follow-up period.

### 2.2. Clinical Data Collection

The demographic and clinical data of the patients were extracted from electronic case report forms in the Femur Fracture Registry. This included information on age, sex, initial blood pressure, heart rate, height, body weight, body mass index (BMI) calculated in kg/m^2^, and medical history including hypertension, diabetes, dyslipidemia, cerebrovascular disease, and atrial fibrillation. The duration of hospital stay was also documented. The laboratory parameters collected included the white blood cell and platelet counts, hemoglobin level, and the serum levels of sodium, potassium, aspartate aminotransferase, alanine aminotransferase, blood urea nitrogen, creatinine, estimated glomerular filtration rate in mL/min/1.73 m^2^, brain natriuretic peptide, creatine kinase-MB, Troponin-I, D-dimer, and C-reactive protein (CRP).

### 2.3. Echocardiographic Parameters and Strain Analysis

TTE images of patients were evaluated. Images were captured using the EPIQ CVx echocardiography system (Philips Medical Systems, Amsterdam, The Netherlands). All echocardiographic assessments were performed by experienced sonographers. Left ventricular (LV) systolic function was assessed by calculating the LV ejection fraction (LVEF) using M-mode images. In cases of reduced LV systolic function, LVEF was determined using the modified Simpson biplane technique. The evaluation of LV diastolic function included measuring the early diastolic transmitral flow velocity (E), early diastolic transmitral flow velocity/early diastolic mitral annular velocity (E/e’) ratio, and pulmonary artery systolic pressure (PASP). PASP values were estimated based on the maximum velocity of the tricuspid regurgitation jet via continuous-wave Doppler and the estimated central venous pressure. RV systolic function was assessed using tricuspid annular plane systolic excursion (TAPSE) and tissue Doppler-derived tricuspid lateral annular systolic velocity (RV s’) [11].

Apical four-chamber view two-dimensional images were gathered for the assessment of longitudinal RV strain, employing an average frame rate of 68 ± 10 frames per second. Images were stored digitally and analyzed offline to ensure consistent and precise measurements. A trained researcher conducted these analyses using the QLAB version 13 software Cardiac Motion Quantification (CMQ) (Philips, Andover, MA, USA), which is an offline program. The process first involved manually outlining the endocardial border of the RV myocardium on an end-systolic frame, followed by automatic delineation of the epicardial border by the software. Manual adjustments were made to accurately align the borders of the areas of interest. Subsequently, the RV myocardium was traced in a frame-by-frame manner, allowing for separate calculation of longitudinal strain in the basal, middle, and apical segments of the RV free wall. The RV peak global longitudinal strain (RV GLS) and RV free wall peak global longitudinal strain (RV free wall GLS) were calculated by averaging these segment-specific values. Given that the length of longitudinal myocardial fibers decreased during systole, this contraction was quantified as a negative value. Therefore, more negative RV GLS values are indicative of enhanced or superior strains. To ensure the accuracy and reproducibility of the strain measurements, each analysis was performed at least twice by the same researcher, and the mean value was used for final analysis. For patients with atrial fibrillation, all RV GLS measurements were averaged over three cardiac cycles. Using the CMQ software (version 13), comprehensive three-dimensional (3D) measurements of the RV were also performed, which included analyses of the RV end-diastolic volume (RVEDV), RV end-diastolic volume index (RVEDVi), RV end-systolic volume (RVESV), RV end-systolic volume index (RVESVi), RV ejection fraction (RV EF) by 3D, and RV stroke volume.

### 2.4. Study Outcomes

The primary outcome was the development of an adverse composite clinical event, which included pneumonia, pulmonary oedema or effusion, pulmonary thromboembolism, and all-cause mortality within the 1-year period following surgery. In addition, the occurrence of this composite event was evaluated at the 1-month mark. Each component of the composite event was individually evaluated both 1 month and 1 year after surgery. The diagnoses of pneumonia, pulmonary oedema, and pleural effusion were made by reviewing medical records and analyzing physical signs along with chest radiography results. CT was used for confirming pulmonary embolism.

### 2.5. Reproducibility of the Strain Analysis

To evaluate the consistency of the strain analysis, another skilled investigator independently reanalyzed 20 randomly chosen images using identical software to determine the interobserver variability of the RV GLS. To assess intraobserver variability, the initial investigator, without knowledge of the previous results, reassessed the RV GLS for each of these randomly chosen images 1 month after the first evaluation. 

### 2.6. Statistical Analyses

In our prospective study of 163 patients, there were no missing data points as all patients completed the follow-up period and provided complete datasets. Categorical data are presented as frequencies and percentages, whereas continuous variables are presented as means with standard deviations. Pearson’s chi-squared test was used to compare categorical variables. For continuous variables with a normal distribution, Student’s *t*-test was applied, whereas the Mann–Whitney U test was used for those with non-normal distribution. The optimal cut-off value of RV GLS for predicting adverse composite clinical outcomes was determined through receiver operator characteristic (ROC) curve analysis. Furthermore, Kaplan–Meier survival analysis and log-rank tests were used to assess the differences in composite event-free survival rates among groups categorized according to the RV GLS cut-off value. To identify potential risk factors for adverse clinical outcomes during follow-up, univariate and multivariate Cox proportional hazards regression analyses were conducted after adjusting for individual risk factors. RV GLS was treated as a dichotomous variable in both univariate and multivariate Cox analyses to verify the consistency of the results based on the determined cut-off value. Variables with a *p*-value < 0.1 in the univariate analysis were chosen for the multivariate model. Interobserver and intraobserver agreements were quantified using the intraclass correlation coefficient (ICC). Statistical significance was set at *p* < 0.05. All statistical analyses were performed using Python version 3.11.5 (Python Software Foundation, Wilmington, DE, USA) and involved libraries such as Pandas 2.1.0, NumPy 1.26.0, SciPy 1.11.2, Lifelines 0.27.8, OpenPyXL 3.1.2, and Matplotlib 3.8.0 for various computational and graphical operations.

## 3. Results

### 3.1. Baseline Characteristics and Clinical Measurements

Table 1 shows the baseline patient characters and clinical data. All patients underwent surgery successfully for femoral fracture during hospitalization, and 36 patients (22.09%) experienced adverse composite clinical events during 1-year follow-up. The baseline patient characteristics are shown in Table 1. There were no significant differences in the clinical characteristics between the adverse and non-adverse outcome groups. However, the adverse outcome group had a significantly longer hospital day (18.53 ± 8.29 vs. 14.16 ± 5.48 days, *p* = 0.002). 

Table 2 shows the results of the laboratory tests and echocardiographic measurements. The adverse outcome group exhibited a significantly lower sodium concentration (134.83 ± 6.17 vs. 137.77 ± 3.88 mmol/L, *p* = 0.003) and a notably higher CRP level (5.74 ± 7.26 vs. 2.07 ± 2.84 mg/dL, *p* <0.001) than the non-adverse outcome group. No significant differences were found in other laboratory tests between the two groups. Regarding cardiac function, the mean LVEF measured via TTE was 63.38 ± 10.09% across all patients. This measure showed no significant differences between the adverse and non-adverse outcome groups. The indices of diastolic function, including E velocity and E/e, showed no significant difference between the groups, as did the indices of RV function, including TAPSE, PASP, and tricuspid insufficiency grades.

### 3.2. Right Ventricular Strain Analysis and 3D Measurement

The strain analysis revealed that the average RV GLS (−13.73 ± 5.7%) and RV free wall GLS (−16.76 ± 7.18%) (Table 3) across all evaluated patients were notably lower than the established normal ranges for a healthy population (RV GLS, −24.5 ± 3.8%; RV free wall GLS, −28.5 ± 4.8%) [12]. Moreover, a significant difference was observed between the adverse and non-adverse outcome groups. The adverse outcome group demonstrated poorer RV GLS and RV free-wall GLS values than did the non-adverse outcome group. Segmental analysis of the strain values revealed that the RV apicolateral wall strain tended to be more impaired in the adverse outcome group. However, the RV volume values, RVEF, and stroke volume evaluated using 3D measurements showed no significant differences between the two groups. 

### 3.3. Development of Adverse Clinical Outcomes

Among 163 patients, 36 patients (22.09%) experienced adverse composite clinical events during 1-year follow-up (mean 319.05 ± 223.58 days, median 324 days). Details of these events are shown in Table 4.

**Figure 2:**
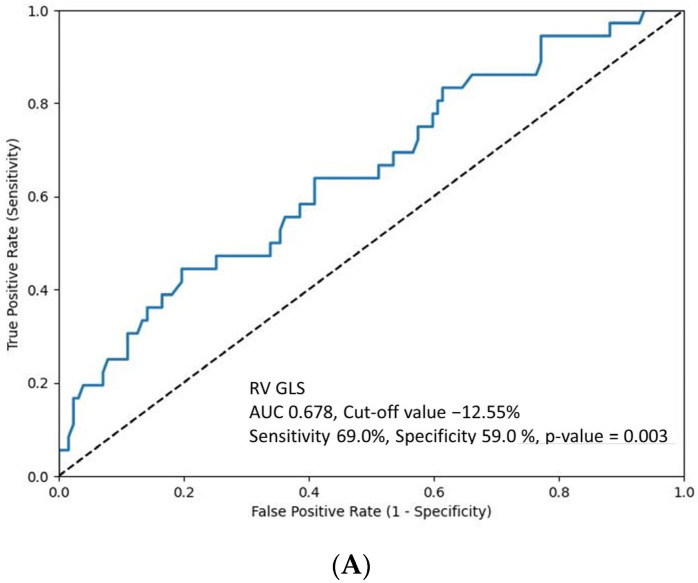

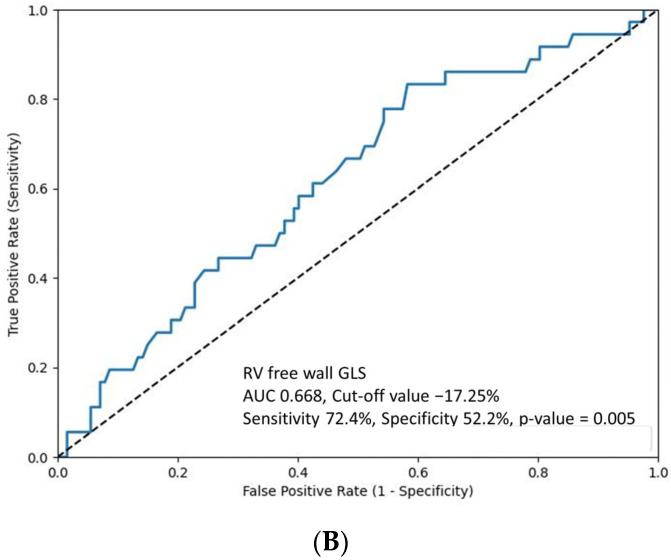
Receiver operating characteristic curve. (**A**) Optimal cut-off value of RV GLS for predicting adverse composite clinical outcomes in patients with femoral fracture. (**B**) Optimal cut-off value of RV free wall GLS. RV GLS, right ventricular global longitudinal strain.

**Figure 3:**
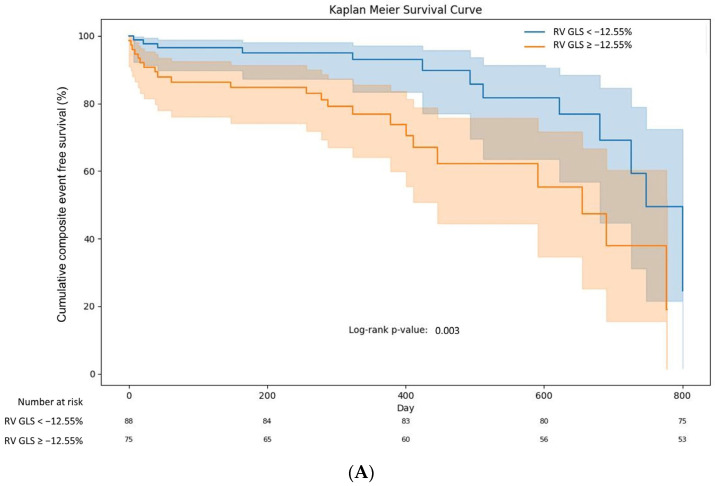

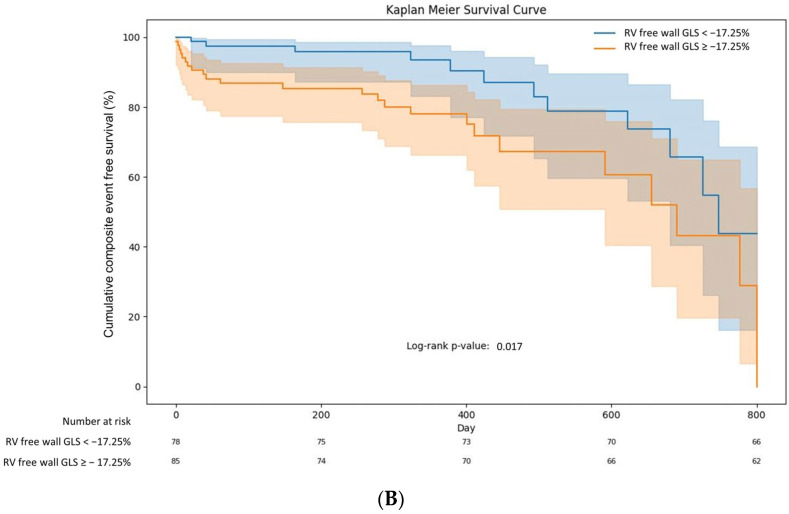
Kaplan–Meier survival curve. (**A**) Cumulative composite event-free survival rate according to RV GLS. (**B**) Cumulative composite event-free survival rate according to RV free wall GLS. The background colors in the Kaplan-Meier survival curve represent the 95% confidence intervals for each group’s survival estimates. RV GLS right ventricular global longitudinal strain.

Figure 2 presents the ROC curve to determine the optimal cut-off values of RV GLS (Figure 2A) and RV free wall GLS (Figure 2B) for predicting composite adverse clinical events at 1-year follow-up. Furthermore, the study examined the incidence of adverse clinical events in the groups stratified by these cut-off values (Table 4). The group with RV GLS ≥ −12.55% experienced a significantly higher rate of both 1-month and 1-year composite adverse clinical events than the group with RV GLS < −12.55%. Notably, among the various adverse clinical events, pulmonary thromboembolism demonstrated a significant difference at both 1-month and 1-year follow-ups. Additionally, the cumulative composite event-free survival rate was significantly lower in the RV GLS ≥ −12.55% (Figure 3A) and RV free wall GLS ≥ −17.25% groups (Figure 3B).

### 3.4. Predictor of Adverse Clinical Events in Patients with Femoral Fracture

Univariate analysis (Table 5) in patients with femoral fracture showed that the following factors were associated with composite adverse clinical events after 1-year follow-up: RV GLS ≥ −12.55%, presence of atrial fibrillation, BMI ≥ 25 kg/m^2^, lower sodium concentration, and RVESVi. After adjusting for confounding factors, multivariate Cox proportional hazards regression analyses showed that RV GLS ≥ −12.55% independently increased the risk of composite adverse clinical events by 2.65-fold (hazard ratio [HR] 2.65, 95% confidence interval [CI] 1.225–5.710, *p* = 0.013). Higher BMI (≥25 kg/m^2^), lower sodium concentration, and higher RVESVi were also shown to independently increase the risk of composite adverse clinical events.

### 3.5. Reproducibility

The reproducibility of the RV GLS measurements demonstrated a high reliability. The interobserver agreements assessed using ICC were quantified as 0.986, and the 95% CI ranged from 0.978 to 0.991 in a retrospective evaluation. The intraobserver agreement was quantified as 0.981, and the 95% CI extended from 0.978 to 0.995.

## 4. Discussion

In our study of 163 patients hospitalized for femoral fractures, we identified a crucial association between diminished RV GLS and a greater risk of adverse clinical outcomes, including pneumonia, pulmonary oedema, thromboembolism, and all-cause mortality, over 1 year. The cut-off value of RV GLS for predicting these events was −12.55% which was determined through ROC curve analysis. Importantly, patients with an RV GLS ≥ −12.55% experienced substantially more adverse events in both 1-month and 1-year follow-up periods than those with an RV GLS below this threshold. Furthermore, these patients had a significantly lower event-free survival rate during the study period. In terms of risk factors, an RV GLS of ≥−12.55% independently increased the risk of adverse clinical outcomes by 2.65-fold. Additional independent risk factors for adverse outcomes included a higher BMI (≥25 kg/m^2^), lower sodium levels, and increased RVESVi.

The observed association between a reduced RV GLS and adverse clinical outcomes in patients with femoral fractures requires consideration of the underlying pathophysiological mechanisms. Acute long bone fractures, such as femoral fractures, can trigger a systemic inflammatory response, which may affect cardiac function [13,14]. This response often leads to an increase in metabolic demand and a subsequent rise in cardiac output, placing additional strain on the RV [13]. Furthermore, the presence of bone marrow and fat emboli, commonly associated with such fractures, can lead to pulmonary embolism or fat embolism syndrome, directly affecting RV function [15,16]. In patients with subclinical RV dysfunction, as indicated by reduced RV GLS, these hemodynamic changes can precipitate decompensation, leading to complications such as pulmonary oedema, thromboembolism, and even mortality. The strain on the RV may be further exacerbated in the presence of pre-existing conditions such as hypertension, atrial fibrillation, or diabetes, which were prevalent in our study cohort. The significant hemodynamic burden imposed by both acute fractures and underlying cardiac dysfunction likely contributes to the higher incidence of adverse outcomes observed in patients with reduced RV GLS. Additionally, systemic inflammation and embolic events can lead to right ventricular remodeling and fibrosis, impairing RV function and contributing to adverse outcomes. Understanding these mechanisms is crucial for developing targeted interventions and improving prognostic assessment in this patient population.

The study also identified a higher BMI (≥25 kg/m^2^) and lower sodium levels as independent risk factors for adverse outcomes. Elevated BMI is often associated with increased cardiovascular risk and may exacerbate the strain on the RV due to higher metabolic demands and the presence of comorbid conditions such as hypertension and diabetes [17,18]. Low sodium levels can indicate underlying health issues such as heart failure or chronic kidney disease, which may contribute to poorer outcomes by exacerbating fluid retention and increasing the cardiac workload [19]. Addressing these factors in clinical practice, alongside RV GLS, could help to provide better risk stratification and management of patients with femoral fractures.

Several animal experimental studies have provided insight into the relationship between long bone fractures and cardiac function. Weber et al. [13] performed a comprehensive analysis of systemic inflammatory responses and their impact on the cardiac tissue after fracture in various subjects, including mice, pigs, and humans. This study encompassed a broad range of subjects using enzyme-linked immunosorbent assays to analyze complement component 5a (C5a), tumor necrosis factor (TNF), and extracellular histones. Key findings included elevated TNF, C5a, and extracellular histones after long bone fractures, along with increased systemic troponin I levels and structural changes in connexin 43 and desmin. These findings on complement activation and systemic inflammation may explain, in part, our study results on RV subclinical dysfunction and help to explain the structural cardiac changes that may occur after femoral fracture. Another study by Weber et al. [20] on pigs with femoral fractures revealed early myocardial damage and valvular dysfunction marked by elevated troponin I and heart-type fatty acid-binding protein levels. The study noted changes in systolic and diastolic heart function as well as pulmonary and tricuspid valve dysfunction. This study highlights the significant impact of long bone fractures on cardiac health, particularly in relation to systolic, diastolic, and valvular functions, reinforcing the importance of assessing RV GLS in patients with femoral fractures. However, to the best of our knowledge, no study has specifically examined RV dysfunction in humans after femoral fractures and its correlation with cardiac and clinical outcomes.

Our study offers vital insights into the care of patients with femoral fractures, highlighting the importance of evaluating RV subclinical function as a part of their clinical assessment. This approach represents a pivotal shift in the evaluation and management of these patients and potentially improves their outcomes. The strengths of our study lie in its prospective design and robust follow-up period, which allowed for a comprehensive analysis of RV function and its impact over time. This study adds a valuable dimension to the existing literature and highlights the need for careful cardiac monitoring in patients with femoral fractures. This opens up avenues for future research exploring targeted interventions that can mitigate the risk of adverse outcomes in this patient population.

However, the limitations of our study should be considered when interpreting these findings. First, as this was a single-center study, the results might not be broadly applicable across diverse patient demographics and clinical practices, as they can vary widely among different institutions and regions. Multicenter trials are planned to validate and extend our findings across a more diverse population and clinical settings. Second, there was a risk of selection bias as the study only included patients who were able to undergo TTE and provided informed consent, potentially omitting those with more severe medical conditions. Additional research should stratify analyses by demographic and clinical characteristics to determine if the predictive value of RV GLS varies across different subgroups. Finally, the observational design of our study means that causality cannot be definitively established between the RV GLS and adverse outcomes, indicating the need for more comprehensive prospective randomized studies to validate and extend our findings. Studies with extended follow-up periods are warranted to explore long-term trends and outcomes, which could provide further insights into the chronic impact of subclinical right ventricular dysfunction in patients with femoral fractures. While our primary analyses are thorough, incorporating sensitivity analyses in future research would further validate the predictive value of RV GLS under various conditions. Moreover, despite high interobserver and intraobserver agreement, potential measurement variability in echocardiographic assessments might still impact the results. Addressing these aspects in subsequent studies will enhance the reliability and generalizability of our findings.

## 5. Conclusions

In conclusion, our study demonstrates that an RV GLS ≥ −12.55% is a significant predictor of adverse clinical outcomes in patients with femoral fractures. This finding underscores the importance of RV GLS in evaluating the risk of complications, such as pneumonia, pulmonary oedema, thromboembolism, and mortality within 1 year of fracture. Additionally, our results highlight the need for clinicians to consider other independent risk factors, such as high BMI, low sodium concentration, and increased RV end-systolic volume, when assessing patient prognosis. Overall, incorporating RV GLS into routine clinical assessments may enhance the prediction and management of adverse outcomes in patients with femoral fractures.

## Figures and Tables

**Table 1:** Baseline characteristics of the patients.

Clinical Variables	All (n = 163)	Adverse Outcome Group (n = 36)	Non-Adverse Outcome Group (n = 127)	*p*
Age, years	81.39 ± 7.94	81.44 ± 10.62	79.86 ± 7.13	0.364
Men, n (%)	45 (27.61)	12 (33.33)	33 (25.98)	0.510
SBP, mmHg	129.07 ± 21.63	132.39 ± 22.64	128.23 ± 19.69	0.331
DBP, mmHg	73.39 ± 12.55	75.56 ± 17.98	73.33 ± 9.7	0.408
HR, beats/min	80.24 ± 13.28	81.58 ± 15.69	79.68 ± 12.38	0.497
Height, cm	157.31 ± 8.92	158.06 ± 8.61	156.7 ± 9.75	0.483
Weight, kg	56.14 ± 11.59	59.64 ± 12.05	56.95 ± 11.95	0.277
BMI, kg/m^2^	22.58 ± 3.75	23.69 ± 3.38	23.11 ± 4.07	0.467
Hypertension, n (%)	124 (76.07)	26 (72.22)	98 (77.17)	0.695
Diabetes, n (%)	75 (46.01)	16 (44.44)	59 (46.46)	0.981
Dyslipidemia, n (%)	35 (21.47)	5 (13.89)	30 (23.62)	0.305
Cerebrovascular disease, n (%)	35 (21.47)	6 (16.67)	29 (22.83)	0.572
Atrial fibrillation, n (%)	12 (7.36)	5 (13.89)	7 (5.51)	0.181
Hospital stay, days	15.68 ± 6.86	18.53 ± 8.29	14.16 ± 5.48	0.002

BMI, body mass index; DBP, diastolic blood pressure; HR, heart rate; SBP systolic blood pressure.

**Table 2:** Laboratory tests and echocardiographic measurements.

	All (n = 163)	Adverse Outcome Group (n = 36)	Non-Adverse Outcome Group (n = 127)	*p*
**Laboratory parameters**				
Hemoglobin, g/dL	10.74 ± 1.86	10.7 ± 1.69	10.96 ± 1.77	0.480
WBC, /mm^3^	8601.53 ± 3039.74	9544.44 ± 3886.76	8363.01 ± 2506.35	0.061
Platelet, 10^3^/μL	191.82 ± 73.15	188.86 ± 81.83	183.12 ± 65.72	0.697
Na, mmol/L	137.0 ± 4.38	134.83 ± 6.17	137.77 ± 3.88	0.003
K, mmol/L	4.03 ± 0.57	3.94 ± 0.6	4.08 ± 0.53	0.218
AST, U/L	31.63 ± 25.22	31.86 ± 14.29	33.45 ± 34.65	0.794
ALT, U/L	20.21 ± 23.76	20.42 ± 26.53	22.84 ± 27.99	0.670
BUN, mg/dL	24.79 ± 17.2	24.74 ± 14.7	23.48 ± 17.92	0.717
Creatinine, mg/dL	1.43 ± 1.74	1.4 ± 1.45	1.39 ± 1.85	0.972
eGFR, mL/min/1.73 m^2^	65.23 ± 29.27	62.26 ± 30.66	68.79 ± 27.71	0.272
BNP, pg/mL	244.43 ± 374.61	280.87 ± 281.15	202.89 ± 360.24	0.262
CKMB, ng/mL	5.2 ± 18.67	11.49 ± 37.98	3.72 ± 6.05	0.094
Troponin-I, μg/L	0.05 ± 0.27	0.02 ± 0.05	0.02 ± 0.09	0.610
D-dimer, ng/mL	7439.51 ± 11,057.0	10,120.47 ± 14,258.04	8043.18 ± 10,494.52	0.396
CRP, mg/dL	3.81 ± 5.76	5.74 ± 7.26	2.07 ± 2.84	<0.001
**Echocardiographic parameters**				
LVEF, %	63.38 ± 10.09	62.3 ± 11.15	63.47 ± 9.34	0.569
LV EDD, mm	49.9 ± 5.2	50.2 ± 5.3	49.8 ± 5.1	0.620
E, cm/s	64.51 ± 22.85	71.71 ± 31.62	62.6 ± 18.83	0.066
E/A ratio	0.75 ± 0.68	0.74 ± 0.54	0.8 ± 0.9	0.686
E/e’	14.18 ± 5.96	14.84 ± 8.56	14.25 ± 4.89	0.650
RV s’, cm/s	13.32 ± 3.37	13.15 ± 2.95	13.42 ± 3.74	0.703
TAPSE, mm	22.77 ± 4.22	22.37 ± 3.31	22.7 ± 4.81	0.710
PASP, mmHg	30.55 ± 10.8	30.36 ± 10.75	29.34 ± 9.83	0.624
Tricuspid insufficiency				0.985
Trivial	67 (41%)	14 (40%)	53 (42%)	
Mild	55 (34%)	13 (35%)	43 (34%)	
Moderate	31 (19%)	7 (20%)	23 (18%)	
Severe	10 (6%)	2 (5%)	8 (6%)	

ALT, alanine aminotransferase; AST, aspartate aminotransferase; BUN, blood urea nitrogen; CKMB, creatine kinase MB; CRP, C-reactive protein; E, early diastolic transmitral flow velocity; E/A, early diastolic transmitral flow velocity/atrial diastolic transmitral flow velocity; E/e’, early diastolic transmitral flow velocity/early diastolic mitral annular velocity; eGFR, estimated glomerular filtration rate; LVEF, left ventricular ejection fraction; PASP, pulmonary artery systolic pressure; BNP, brain-type natriuretic peptide; RV, right ventricle; TAPSE, tricuspid annular plane systolic excursion; WBC, white blood cell.

**Table 3:** RV strain analysis and 3D RV measurements.

	All (n = 163)	Adverse Outcome Group (n = 36)	Non-Adverse Outcome Group (n = 127)	*p*
RV GLS (%)	−13.73 ± 5.7	−11.38 ± 5.43	−14.2 ± 5.62	0.015
RV free wall GLS (%)	−16.76 ± 7.18	−14.47 ± 6.79	−17.49 ± 7.34	0.043
Basal lateral	−20.87 ± 8.89	−18.87 ± 9.22	−21.53 ± 8.87	0.153
Mid lateral	−16.23 ± 7.04	−14.48 ± 7.61	−16.8 ± 6.92	0.118
Apicolateral	−13.18 ± 7.57	−10.75 ± 7.03	−13.81 ± 7.94	0.054
RVEDV, mL	89.49 ± 31.21	92.62 ± 32.22	86.8 ± 29.91	0.358
RVEDVi, mL/m^2^	55.77 ± 17.28	57.24 ± 17.95	54.46 ± 16.23	0.423
RVESV, mL	55.26 ± 20.76	58.77 ± 22.93	51.95 ± 17.28	0.089
RVESVi, mL/m^2^	34.39 ± 11.53	36.28 ± 13.06	32.54 ± 9.09	0.089
RV EF by 3D, %	38.28 ± 9.33	36.65 ± 12.5	39.69 ± 6.99	0.110
RV stroke volume (mL)	35.59 ± 23.36	33.93 ± 16.45	37.89 ± 30.54	0.472

RVEDV, right ventricular end-diastolic volume; RVEDVi, right ventricular end-diastolic volume index; RVESV, right ventricular end-systolic volume; RVESVi, right ventricular end-systolic volume index; RVEF, right ventricular ejection fraction; RV GLS, right ventricular global longitudinal strain.

**Table 4:** Adverse clinical event rate according to RV strain.

Clinical Variables	All (n = 163)	RV GLS ≥ −12.55 (n = 75)	RV GLS < −12.55 (n = 88)	*p*
1-month composite event	29 (17.79)	20 (26.67)	9 (10.23)	0.011
1-month pneumonia	10 (6.13)	6 (8.0)	4 (4.55)	0.515
1-month pulmonary oedema or effusion	13 (7.98)	7 (9.33)	6 (6.82)	0.764
1-month PTE	4 (2.45)	4 (5.33)	0 (0.0)	0.043
1-month all-cause death	8 (4.91)	6 (8.0)	2 (2.27)	0.145
1-year composite event	36 (22.09)	23 (30.67)	13 (14.77)	0.025
1-year pneumonia	11 (6.75)	6 (8.0)	5 (5.68)	0.783
1-year pulmonary oedema or effusion	16 (9.82)	9 (12.0)	7 (7.95)	0.548
1-year PTE	4 (2.45)	4 (5.33)	0 (0.0)	0.043
1-year all-cause death	12 (7.36)	8 (10.67)	4 (4.55)	0.228

PTE, pulmonary thromboembolism; RV GLS, right ventricular global longitudinal strain.

**Table 5:** Univariate and multivariate analysis of RV GLS for predicting adverse composite outcomes.

	Univariate			Multivariate		
	HR	95% CI	*p*	HR	95% CI	*p*
RV GLS ≥ −12.55%	2.80	1.388–5.648	0.004	2.65	1.225–5.710	0.013
Atrial fibrillation	3.62	1.338–9.769	0.011	1.92	0.622–5.916	0.256
BMI ≥ 25 kg/m^2^	2.80	1.416–5.531	0.003	3.90	1.858–8.182	<0.001
Na, mmol/L	0.95	0.903–0.999	0.044	0.92	0.869–0.984	0.014
BNP, pg/mL	1.00	0.999–1.001	0.070	1.00	0.999–1.001	0.657
CRP, mg/dL	1.04	0.999–1.085	0.053	1.04	0.990–1.095	0.116
RVESVi, mL/m^2^	1.03	1.000–1.061	0.046	1.04	01.004–1.071	0.026

BMI, body mass index; BNP, brain natriuretic peptide; CI, confidence interval; CRP, C-reactive protein; HR, hazard ratio; RVESVi, right ventricular end-systolic volume index; RV GLS, right ventricular global longitudinal strain.

## Data Availability

The data presented in this study are available on request from the corresponding author. The data are not publicly available due to privacy restrictions.
